# Relating Bryophyte Assemblages to a Remotely Sensed Depth-to-Water Index in Boreal Forests

**DOI:** 10.3389/fpls.2018.00858

**Published:** 2018-06-25

**Authors:** Samuel F. Bartels, Richard T. Caners, Jae Ogilvie, Barry White, S. Ellen Macdonald

**Affiliations:** ^1^Department of Renewable Resources, University of Alberta, Edmonton, AB, Canada; ^2^Royal Alberta Museum, Edmonton, AB, Canada; ^3^Nexfor-Bowater Forest Watershed Research Centre, Faculty of Forestry and Environmental Management, University of New Brunswick, Fredericton, NB, Canada; ^4^Forest Management Branch, Alberta Ministry of Agriculture and Forestry, Edmonton, AB, Canada

**Keywords:** boreal forest, depth-to-water index, mosses, liverworts, moisture gradient, site wetness, wet-areas mapping

## Abstract

Given the habitat moisture (air humidity or soil moisture) preferences of many forest bryophytes, we explored whether the depth-to-water (DTW) index, derived from remotely sensed Light Detection and Ranging (LiDAR) data, was related to fine-scale patterns of spatial variation in bryophyte abundance, diversity, and composition. The goal was to assess the utility of the topographic DTW index as a tool to decipher trends in bryophyte assemblages along a site wetness gradient in the boreal mixedwood forest. Discrete Airborne Laser Scanning (ALS) data were acquired over the entire Ecosystem Management Emulating Natural Disturbance (EMEND) experimental site located in northwestern Alberta, Canada (56° 46′ 13″ N, 118° 22′ 28″ W), based on which we calculated a mathematical index of approximate depth to water at or below the soil surface at 1 m resolution using the Wet-Areas Mapping model. Bryophytes (mosses and liverworts) were sampled in permanent sample plots in unmanaged forest stands of varying dominant canopy tree composition. The relationships between DTW and bryophyte cover, richness, diversity, and composition in broadleaf (deciduous)-, mixed, and conifer-dominated boreal forest stands were analyzed using linear mixed-effect models and multivariate analyses. Bryophyte cover was highest in conifer-dominated forest, which occupied the wetter end of the DTW gradient, followed by mixed forest, whereas broadleaf forest, which occupied the drier end of the DTW gradient, had the lowest cover but highest bryophyte diversity. Bryophyte cover in conifer-dominated forests was positively related to site moisture (negatively related to the DTW index). In contrast, bryophyte species richness and diversity were negatively related to site moisture (increased at higher DTW values) in all forest types. DTW explained significant variation in bryophyte species composition in mixed forests, while indicator species analysis identified species with preferences for wet, moist, and dry site conditions in each forest type. Our results corroborate the importance of site moisture as a driver of bryophyte assemblages but, interestingly, there were important differences among forest types, which themselves are distributed across a gradient of site moisture. Our study demonstrates the utility of the topographic DTW index for understanding fine-scale (plot-level) variation in bryophyte assemblages in forested landscapes.

## Introduction

Knowledge of species–habitat associations is central to the design and implementation of effective habitat management plans and conservation strategies (Hannah et al., 2002; Wilhere, 2002). In forest ecosystems, site environmental factors, such as soil moisture, often strongly influence patterns of understory plant composition and diversity (Hutchinson et al., 1999; Brosofske et al., 2001; North et al., 2005). Forest-dwelling bryophytes grow across a variety of substrate types that are characterized in part by differences in moisture conditions (Bates, 1998). For many forest bryophytes, the availability of moisture determines the success of critical life history stages (Wiklund and Rydin, 2004) and can influence the growth and persistence of species within a site (Mills and Macdonald, 2004, 2005; Caners et al., 2013).

The moisture content of bryophytes is at equilibrium with the surrounding air or substrate humidity. Although they are known to be desiccation tolerant (Alpert, 2006), many bryophyte species are negatively affected by desiccation and evaporative stress where moisture is deficient (Busby et al., 1978; Proctor, 2000). Many bryophyte species that grow in closed-canopy forests are favored by moist or wet (both air humidity and soil moisture) habitat conditions (Frisvoll and Prestø, 1997; Berg et al., 2002; Caners et al., 2013). Adequate moisture supply is essential to the productivity and ecological functions of bryophytes in forest ecosystems, which include carbon and nitrogen cycling, mineral acquisition and nutrient retention, and regulation of soil thermal and hydrological regimes (Bates, 1992; Turetsky, 2003; Soudzilovskaia et al., 2013). Terrain characteristics such as topography influence site moisture (through both ground water and surface flows) and, therefore, represent a potentially important environmental control of bryophyte diversity and composition (Dynesius et al., 2009; Kuglerova et al., 2016).

Topography is a major determinant of site moisture because of its pervasive influence on hydrology (Schor and Gray, 2008). Recent studies have shown how topography, through its influence on soil moisture and edaphic properties, can explain variation in patterns of plant diversity and composition (Zinko et al., 2005; Moeslund et al., 2013a,b, and also reviewed in Moeslund et al., 2013c). However, existing studies on the extent to which topography and related attributes determine plant diversity and distribution patterns are challenged by the limited spatial extent of surveys due to difficulties in accessing adequate topographic data across large areas. Local vegetation, including different tree species (e.g., evergreen conifer vs. deciduous broadleaf trees) that differ in rooting system and water consumption, can also influence soil hydrological properties, such as runoff, water table, and soil water content (Jost et al., 2012). This can have direct effects on the abundance and composition of bryophyte species growing beneath the dominant tree canopy.

Recent advances in remote sensing, such as Light Detection and Ranging (LiDAR) technology, enable the development of highly accurate digital elevation models (DEMs) at fine-scale spatial resolution (Murphy et al., 2008; Vierling et al., 2008). DEMs obtained from LiDAR offer significant improvements and accuracy of topographic details over conventional DEMs, and have variously been used to map and predict wet areas on the landscape and soil physical and chemical properties including moisture content, drainage, and soil type (Murphy et al., 2007, 2009, 2011; White et al., 2012). LiDAR-based DEMs have also been used to derive secondary topographic indices, such as the topographic wetness index (TWI) and the newly developed depth-to-water (DTW) index, which provides an approximation of depth to water at or below the soil surface (Murphy et al., 2008; White et al., 2012). There is evidence that the DTW index performs better than the widely used TWI, and could improve mapping of wet areas in the boreal landscape (Murphy et al., 2011; Ågren et al., 2014). While the DTW index was originally conceived and developed as an operational tool to guide forest operations it has been used as a fine scale predictor of ecosystem function and productivity (Oltean et al., 2016; Bjelanovic et al., 2018). It is also well suited to differentiating vegetation attributes such as forest cover-types (Nijland et al., 2015). The operational capabilities of the DTW index, from a management perspective, makes it a useful tool for demarcating and prioritizing potential areas of conservation interest such as wet areas and even biodiversity hotspots on the landscape.

Our goal in this study was to investigate whether terrain information based on remote sensing LiDAR technology can be used to predict and decipher trends in fine-scale (plot-level) bryophyte assemblages along a site wetness gradient in the boreal mixedwood forest. Specifically, we (1) evaluated the relationship of DTW, as a hydrologic index, to bryophyte abundance, richness, diversity, and composition in unmanaged boreal forest stands. Given the strong influence of canopy trees on insolation and humidity in the understory (Barbier et al., 2008), and therefore risks of desiccation and radiation damage in bryophytes, we further examined (2) whether the relationships between DTW and bryophyte assemblages differ across three dominant forest types (broadleaf-, mixed, and conifer-dominated forests) in the boreal mixedwood forest. These forest types are known to occupy different (drier to wetter, respectively) positions along the topographic moisture gradients on the landscape (Nijland et al., 2015), while also supporting different bryophyte assemblages (Bartels et al., 2018). We expected higher bryophyte abundance and diversity and different species composition at the wetter end of the moisture gradient (as determined by DTW), as compared to drier sites where conditions favor species tolerant of drier conditions. Additionally, we anticipated that the association between bryophyte assemblages and DTW would vary among the different forest types as a reflection of their differential distribution with respect to DTW gradient or topographic position.

## Materials and Methods

### Study Area

The study took place in the mixedwood boreal forests of western Canada. Field data were collected at the Ecosystem-based Management Emulating Natural Disturbance (EMEND) research site (56° 46′ 13″ N, 118° 22′ 28″ W), which is located in the Clear Hills Upland, Lower Boreal Cordilleran Ecoregion of Alberta (Natural Regions Committee, 2006). The landscape is characterized by relatively mild topography with flat areas interspersed with elevated blocks rising from 677 to 880 m above sea level with incised valleys. The soils are fine-textured Luvisols originating from glacio-lacustrine deposits (Kishchuk, 2004). The mean annual minimum and maximum temperature recorded at the closest meteorological station in Peace River, Alberta are -4.2°C and 7.3°C, respectively, and total annual precipitation is 386.3 mm, with approximately three-quarters falling as rain (Environment and Climate Change Canada, 2017). Mature forest stands (90–120 years old) at EMEND are dominated by trembling aspen (*Populus tremuloides* Michx.), white spruce (*Picea glauca* (Moench) Voss), and balsam poplar (*Populus balsamifera* L.).

EMEND is a large-scale experimental study of variable retention harvesting (detailed description of experiment design can be found at the project website^1^). For the present study, we selected unharvested forest compartments across three forest cover-types: broadleaf (deciduous)-dominated stands (composed of more than 70% basal area of broadleaf canopy trees species); mixed stands (composed of mixed broadleaf and conifer canopy trees with neither making up > 70% of the canopy); and conifer-dominated stands (composed of more than 70% basal area of conifer canopy tree species). These forest types occupy different positions along the topographic moisture gradients of the landscape, with conifer-dominated stands on wetter areas, followed by mixed and then broadleaf-dominated stands on drier sites (Nijland et al., 2015).

### LiDAR Data Acquisition and Determination of the Depth-to-Water Index

Discrete Airborne Laser Scanning (ALS) data were acquired over the entire study area in August 2008 using a Leica ALS50-II sensor flying at a mean altitude of 2000 m above ground. A bare Earth DEM was generated from the ground returns with an average density of 2 pts/m^2^. The generated DEMs were then processed using the wet areas mapping algorithm to calculate DTW as a wetness index characterizing relative moisture gradients across the entire study area at 1 m spatial resolution (details of DEM sources and wet areas mapping processing are described in Nijland et al., 2015).

The DTW index (unit in meters) provides an approximation of depth to water (at or below the soil surface) based on the elevation difference between a given cell (pixel) and a cell that is a source of water (Murphy et al., 2008). It is expressed as follows:

$$DTW=[\Sigma \frac{{dz}_{i}}{{dx}_{i}}a]x_{c}$$

where *dz/dx* is the slope of a cell, *i* represents a cell along the path, *a* is 1 when the path crosses the cell parallel to the cell boundaries and $\sqrt{2}$ when it crosses diagonally, and *x_c_* is grid cell size.

The mathematical function (as described in Murphy et al., 2009, 2011) interpolates the least slope path from each cell in the landscape to the source cell, based on the cumulative value of slopes along the possible paths. Thus, the index reflects both the distance from a source and the slope of the land surface between the landscape cell and the hydrological source. A flow accumulation network, based on the DEM, is then developed using the D8 flow algorithm to determine flow direction. When flow accumulation at a cell has reached the flow-initiation threshold (i.e., the amount of water accumulation needed to start a flowing channel), stream flow is assumed to begin at that cell. Streams and cells with water accumulation above the initial threshold are given a DTW value of zero. Hence, low DTW values indicate wet, poorly to imperfectly drained sites while high values generally indicate dry sites. Although we did not measure actual groundwater level, the accuracy of the DTW index has been extensively tested and validated in the boreal landscape (e.g., Murphy et al., 2011; Ågren et al., 2014), including extensive validation in our study area (White et al., 2012).

**FIGURE 1:**
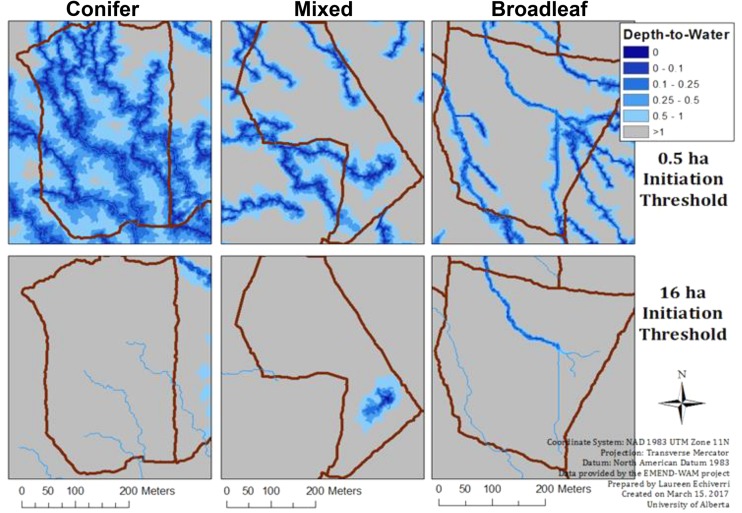
Overview of the depth-to-water gradient (DTW, at flow initiation thresholds λ = 0.5 and 16 ha) from the wet areas mapping (based on airborne laser scanning data) at the EMEND compartment-level, contrasted between conifer- (wetter), mixed (intermediate) and broadleaf-dominated (drier) forests. Darker shades of blue delineate moist sites.

The DTW index is sensitive to the drainage area or flow-initiation threshold (which varied from 0 to 16 ha) used to determine whether a given cell is a source, or not. For instance, DTW based on a low flow-initiation threshold (λ=) of 0.5 ha produces a landscape with more predicted streams (i.e., more areas are predicted to be wet) whereas a high flow-initiation threshold (e.g., 16 ha) produces a more conservative estimate with fewer areas predicted to be wet (see illustration in **Figure 1**). The optimal flow-initiation threshold may depend on the specific application of the wet areas map. For mechanical applications (e.g., road building) the value was determined to be 4 ha in our study area (White et al., 2012). Others also found the 8 ha threshold suitable for differentiating forest cover types in the study area (Nijland et al., 2015). Herein we sought to further test different threshold values to evaluate the capabilities of the DTW index. To determine the optimal flow-initiation threshold suitable for modeling ground cover vegetation attributes at the plot level, we compared the performance of DTW computed with flow-initiation thresholds at six different settings (λ = 0.5, 1, 2, 4, 8, 12, and 16 ha). Each of these was used as the upstream contributing area threshold for the flow channel initiation based on which we calculated DTW index values across the landscape for each threshold.

### Vegetation Sampling

We sampled in three compartments (∼10 ha) per forest type; in each of these we placed eight to 14 sampling locations (total of 111). Sampling locations within each compartment (see Appendix S1) were carefully selected so as to capture the gradient in site moisture using mapped (0.5 ha flow-initiation threshold) DTW index values in ArcGIS v.10.2.1 for Desktop (Environmental Systems Research Institute Inc., Redlands, CA, United States). Sampling locations were then located in the field using a GPS system that was capable of sub-meter accuracy (the SX Blue GPS II, Geneq Inc., Montreal, QC, United States).

Bryophyte (moss and liverwort) species presence and cover were assessed in one 2 m × 2 m quadrat at each sampling location. Individual bryophyte species cover per plot was visually estimated as the vertical projection of ground surface covered by the species. Bryophytes were censused on all surfaces in the 2 m × 2 m plot, including the forest floor, downed woody debris, and the bark and bases of living and non-living woody plants (trees and shrubs), snags, and stumps (all up to 50 cm height). These substrate types or microhabitats were not specifically targeted or controlled for during sampling. Bryophyte species encountered were identified in the field when possible; samples of species that could not be easily identified were brought back to the laboratory for accurate identification. Species nomenclature for mosses follows the Flora of North America (Flora of North America Editorial Committee, 2007, 2014), and that for liverworts follows Stotler and Crandall-Stotler (2017).

### Statistical Analysis

Bryophyte cover per plot was estimated as the sum of the percent cover of individual bryophyte species. Species richness was calculated as the total number of species per plot. Diversity, following Hill numbers (Hill, 1973), was assessed as equivalent Simpson index and estimated as the inverse of Simpson’s index: ($\left[1/\overset{s}{\underset{i=1}{\Sigma }}p_{i}^{2}\right]$ where *p_i_* is the proportional abundance of each species in a sample, estimated on the basis of percent cover). Species composition was examined as the plot-by-species matrix of percent cover values. We tested for significant differences in bryophyte cover, richness, and diversity among the forest types, irrespective of DTW, using one-way analysis of variance, and compared their mean values using least square means. To evaluate the relationship between DTW and bryophyte cover, richness, and diversity we used linear mixed effect models that included DTW as a continuous fixed effect and forest compartment as random (blocking) effect, as follows:

$$Y_{ij}=\mu +{DTW}_{\left(i\right)}+e_{j\left(i\right)}$$

where *Y* is plot-level dependent variable (cover, species richness, or diversity), μ is the intercept, *DTW* is the hydrological DTW index, and, *e* is random error (*j* = compartment).

These models were constructed for each reshold. We compared the predictive capacity of the models and the most supported model (representing the optimal flow-initiation threshold) for each dependent variable was selected based on the second order Akaike Information Criterion (AICc). AICc is desirable when the ratio of the number of observations (*n*) to the number of parameters (*k*) is less than 40 (Burnham and Anderson, 2002). We also calculated the difference between the model with the smallest AICc (AICc*_min_*) and all other models (Δ = AICc*_i_*–AICc*_min_*) to represent the loss of information for the model being compared to the best model. As a rule of thumb, a Δ*_i_* < 2 suggests substantial evidence for the model, values between 3 and 7 indicate considerably less support, whereas Δ*_i_* > 10 indicates that the model is very unlikely (Burnham and Anderson, 2002). Data were transformed as necessary to meet the assumptions of normality and homogeneity of variances. Species richness (i.e., count) data were not transformed, but were analyzed with the Poisson error distribution and a log link function. The linear mixed effect models were performed using the *lmer and glmer* functions in the LME4 package (Bates et al., 2015) with conditional model *R*-squares generated with the MUMIN package (Barton, 2016).

To test for the influence of DTW on species composition, we performed permutational multivariate analysis of variance (PERMANOVA) that included DTW as the explanatory variable. PERMANOVA, which is a non-parametric multivariate analysis that uses permutation techniques, was run (using the *adonis* function in VEGAN) by specifying the Bray-Curtis dissimilarity and 999 permutations of the compositional data. We then examined the trends in the compositional data using non-metric multidimensional scaling (NMDS; Kruskal, 1964) based on the Bray-Curtis dissimilarity index. DTW (continuous variable) was passively displayed as contours in the NMDS ordination using the *ordisurf* function in VEGAN package (Oksanen et al., 2015). Furthermore, we performed Indicator Species Analysis (ISA: Dufrêne and Legendre, 1997) to identify bryophyte species that were associated with wet, moist, or dry site conditions (categorized using the following DTW classes: wet [0–0.5 m], moist [0.6–2 m], and dry [>2 m depth-to-water]) in individual forest types. An evaluation of sampling completeness based on species accumulation curves for these site wetness categories indicated adequate species capture in individual forest types (Appendix S2). ISA was performed in PC-ORD v. 5 (MjM Software Design, Gleneden Beach, OR, United States) with the default settings and the statistical significance of the indicator value (IV) for each species determined through Monte Carlo permutations. All other statistical analyses were performed in R version 3.2.1 (R Core Team, 2015).

## Results

Sampling in each forest type covered a gradient from wet to dry, as indicated by the DTW index values at the different flow-initiation thresholds (**Table 1**). Conifer-dominated forests covered a relatively narrower range of DTW values, which corresponded to the wetter end of the gradient, as compared to those of broadleaf-dominated forests, which were toward the drier end of the gradient. DTW values in mixed forests were intermediate to those of conifer and broadleaf-dominated forests (**Figure 1** and **Table 1**). Bryophyte cover was higher in conifer-dominated (by about 10-fold) and mixed forests than in broadleaf-dominated forest, which had very low cover of bryophytes (**Table 1**). Bryophyte species richness (see species list in Appendix S3) did not differ significantly among the forest types. Bryophyte diversity was lower in conifer-dominated than in broadleaf-dominated forest and was intermediate in mixed forest (**Table 1**).

**Table 1:** The range of depth-to-water (m) index values at different flow-initiation thresholds, covered by our sampling of broadleaf, mixed, and conifer-dominated forests.

Flow initiation threshold	Broadleaf (*n* = 34)	Mixed (*n* = 38)	Conifer (*n* = 39)
			
	Mean (min – max)	Mean (min – max)	Mean (min – max)
Flow-initiation threshold			
0.5 ha	0.89 (0.02 – 4.09)	0.52 (0.01 – 2.76)	0.42 (0.01 – 1.65)
1 ha	1.37 (0.02 – 7.81)	0.74 (0.01 – 4.65)	0.57 (0.01 – 2.71)
2 ha	1.49 (0.02 – 9.48)	1.06 (0.01 – 5.89)	0.57 (0.01 – 2.89)
4 ha	1.76 (0.03 – 11.22)	1.53 (0.01 – 7.27)	0.62 (0.01 – 3.06)
8 ha	3.02 (0.12 – 11.78)	1.61 (0.03 – 7.27)	1.89 (0.01 – 5.57)
12 ha	6.14 (0.12 – 17.01)	1.71 (0.03 – 7.27)	1.93 (0.01 – 5.57)
16 ha	7.38 (0.16 – 17.01)	1.79 (0.03 – 7.27)	2.05 (0.01 – 5.57)
Stand characteristics			
Basal area (m^2^/ha)	34.1 (28.9 – 39.3)	47.7 (42.5 – 52.9)	48.3 (41.6 – 55.0)
^†^Conifer (%)	2.3 (0.5 – 4.1)	63.4 (55.0 – 71.8)	86.2 (81.7 – 90.7)
Bryophyte species attributes			
Cover (%)	2.83 (0 – 17.20)*^a^*	19.79 (1.70 – 79.9)*^ab^*	30.99 (2.30 – 93.20)*^b^*
Richness	10.09 (0 – 21.0)*^a^*	12.74 (2.0 – 33.0)*^a^*	10.92 (3.0 – 32.0)*^a^*
Diversity	5.31 (0 – 13.0)*^b^*	3.47 (1.10 – 17.0)*^ab^*	2.41 (1.10 – 6.29)*^a^*


*Values are mean (min – max) and *n* is number of plots. Also given are overall mean values for bryophyte cover, richness, and diversity for each forest type. ^†^Describes the proportion of basal area (%) represented by conifer tree species (mainly white spruce) in individual forest types. Dominant broadleaf tree species included trembling aspen and balsam poplar. For a given bryophyte attribute, different superscript letters denote significant differences (at α < 0.05) among forest types based on least squared means comparison.*

There was no clear optimal flow-initiation threshold for modeling the relationship between DTW and bryophyte cover, richness, and diversity, as AICc_min_ values differed among the bryophyte response attributes in the different forest types (Appendix S4). Assessment of the change in AICc, however, indicated considerable support (ΔAICc < 2 across all the bryophyte response attributes) for the 4 ha flow-initiation threshold for both broadleaf- and conifer-dominated forest and for the 4, 8, 12, and 16 ha thresholds (which were highly correlated and therefore functional equivalent; see Appendix S5) in mixed forest (Appendix S4). Based on this result we used DTW index computed with the 4 ha flow-initiation in all subsequent analyses. The 4 ha flow-initiation threshold is suggested to coincide with the minimum catchment area for image-digitized or field-mapped stream channels with permanent or intermittent flow (Murphy et al., 2009).

### Depth-to-Water Relationship With Bryophyte Cover, Richness, and Diversity

**FIGURE 2:**
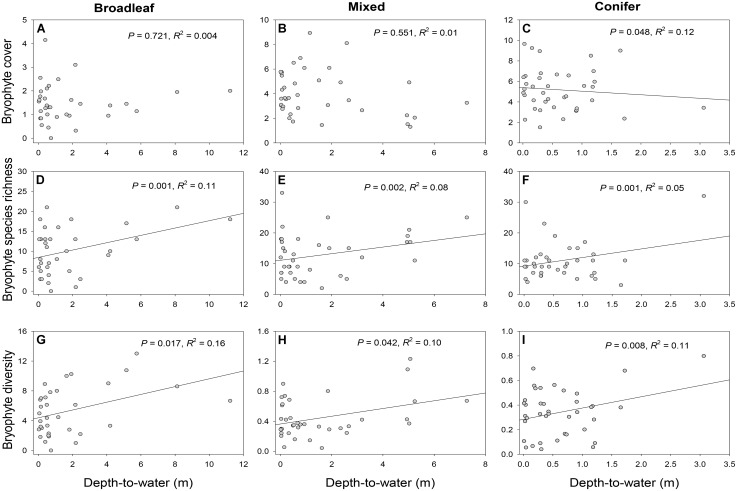
Relationships between depth-to-water (DTW) and bryophyte cover **(A–C)**, richness **(D–F)** and diversity **(G–I)** in broadleaf-, mixed, and conifer-dominated boreal forest stands. DTW was based on the 4 ha flow-initiation threshold which produced the best-supported model (based on model AICc and ΔAICc; see Appendix S2). For the analyses bryophyte cover was square root transformed for all forest types **(A–C)** and bryophyte diversity was log transformed for mixed **(H)** and conifer forests **(I)**. R-squared (*R*^2^) values describe the proportion of variance explained by the fixed factor, i.e., DTW (see Equation 1) alone.

There was no significant relationship between bryophyte cover and DTW for broadleaf-dominated (*P* = 0.721, *R*^2^ = 0.004) or mixed (*P* = 0.551, *R*^2^ = 0.01) forests (**Figures 2A,B** and **Table 2**). However, in conifer-dominated forest bryophyte cover was positively related to wetness (*P* = 0.048, *R*^2^ = 0.12) (i.e., cover decreased with increasing DTW) (**Figure 2C** and **Table 2**). Bryophyte species richness in all three forest cover-types was negatively related to wetness (i.e., richness increased with increasing DTW) (**Figures 2D–F** and **Table 2**). Similarly, bryophyte diversity in broadleaf-dominated (*P* = 0.017, *R*^2^ = 0.16), mixed (*P* = 0.042, *R*^2^ = 0.10) and conifer-dominated (*P* = 0.008, *R*^2^ = 0.11) forests was negatively related to wetness (i.e., increased with increasing DTW) (**Figures 2G–I** and **Table 2**).

**Table 2:** Relationship between depth-to-water (DTW, based on the 4 ha flow-initiation threshold) and bryophyte cover, richness, and diversity in three boreal forest-cover types.

Forest type	Response variable	μ	Depth-to-water (DTW)				*R*^2¥^
				
			Coef.	*SE*	*t*-value	*P*-value	
Broadleaf forest	Cover^†^	1.45	0.02	0.06	0.360	0.721	0.01
	Richness	2.17	0.07	0.02	3.812	<0.001	0.12
	Diversity	4.39	0.52	0.21	2.519	0.017	0.16
Mixed forest	Cover^†^	4.04	-0.08	0.14	-0.603	0.551	0.43
	Richness	2.43	0.07	0.02	3.111	0.002	0.08
	Diversity^§^	0.88	0.10	0.05	2.109	0.042	0.24
Conifer forest	Cover^†^	6.23	-0.54	0.25	-2.156	0.038	0.15
	Richness	2.24	0.22	0.07	3.192	0.001	0.07
	Diversity^§^	0.64	0.25	0.09	2.833	0.008	0.51


*^¥^*R*^*2*^describes the proportion of variance explained by both the fixed and random factors (*sensu*Nakagawa and Schielzeth, 2013). ^†^Indicates square root transformation of dependent variable. ^§^Indicates natural log transformation of dependent variable.*

### Influence of Depth-to-Water on Bryophyte Species Composition

**FIGURE 3:**
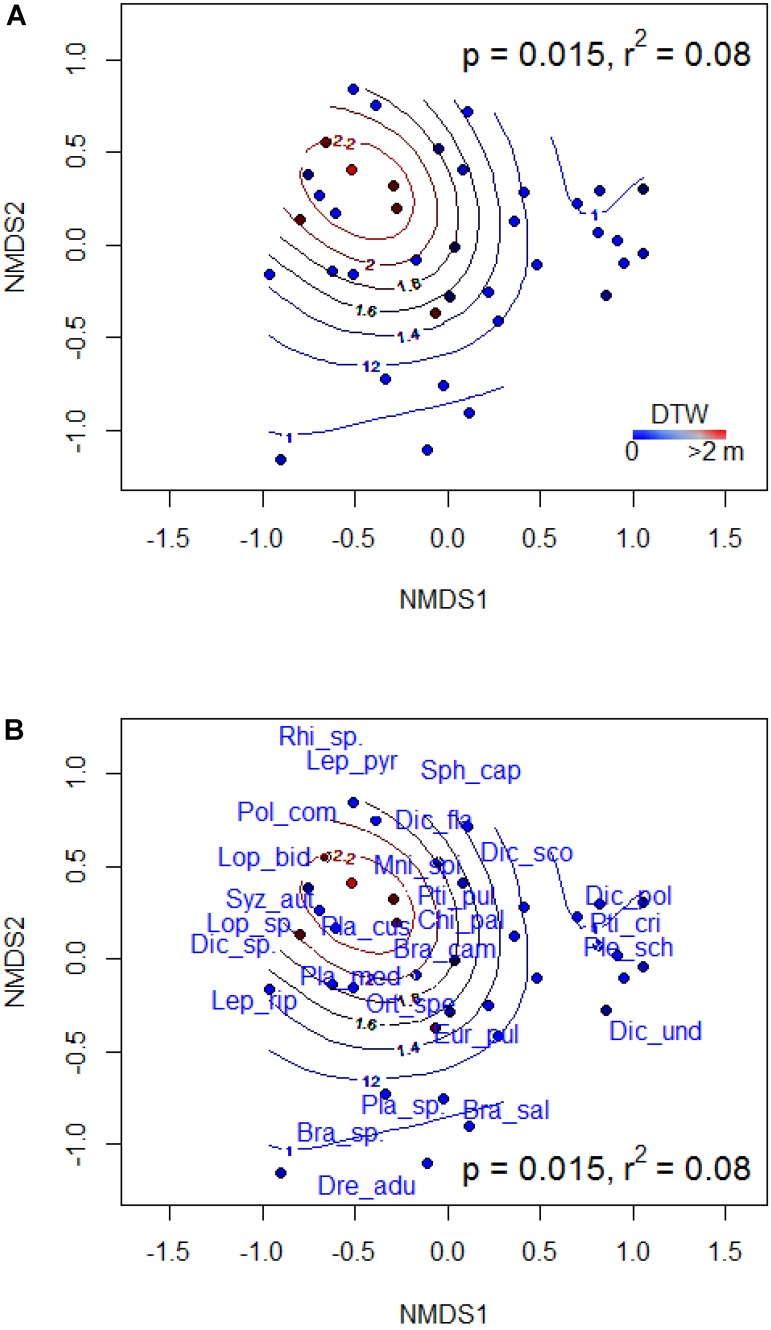
Non-metric multidimensional scaling (NMDS) ordination of bryophyte species composition in mixed forest showing the distribution of sites **(A)** and species **(B)**. Best NMDS solution was reached at a stress of 0.228. Contours display the depth-to-water gradient. *P*-values indicate the statistical significance and *R*^2^ values describe the proportion of variance in species composition explained by DTW. Species list and codes are provided in Appendix S3. The abbreviations NMDS1 and NMDS2 denote the first (i.e., axis 1) and second (i.e., axis 2) axes, respectively, of the NMDS ordination.

In broadleaf-dominated forest which occupied the drier end of the gradient, the results of PERMANOVA indicated that bryophyte species composition did not vary significantly with DTW (*F* = 1.294, *P* = 0.195, *R*^2^ = 0.04). Indicator species in broadleaf forest were all associated with dry site conditions, and these included: *Campylophyllum hispidulum*, *Chiloscyphus pallescens*, *Lophocolea heterophylla*, *Plagiomnium drummondii*, and *Scapania glaucocephala* (**Table 3**). In mixed forest, bryophyte species composition varied significantly with DTW (*F* = 2.934, *P* = 0.015, *R*^2^ = 0.08) with plots distributed along the DTW gradient (**Figure 3A**). Some indicator species of wet and moist site conditions in mixed forest included *Pleurozium schreberi*, *Hylocomium splendens*, *Aulacomnium palustre*, and *Thuidium recognitum*. Others such as *Brachythecium campestre*, *Orthotrichum speciosum*, *Pylaisia polyantha*, and *Plagiomnium drummondii* were indicators of dry site conditions (**Figure 3B** and **Table 3**). In conifer-dominated forests, which occupied the wetter end of the gradient, DTW did not explain significant variation in bryophyte species composition (*F* = 1.719, *P* = 0.116, *R*^2^ = 0.04). Indicator species of wet and moist site conditions in conifer forest included the liverwort *Ptilidium pulcherrimum* and the mosses *Dicranum fragilifolium* and *Thuidium recognitum* (**Table 3**).

**Table 3:** Bryophyte species that were indicators of site wetness (wet [0–0.5 m], moist [0.6–2 m], and dry [>2 m depth-to-water]) in broadleaf, mixed, and conifer forests.

Forest type	Site wetness	Indicator species	Indicator value	Indicator values from randomization		
				
				Mean	*SD*	*P*
Broadleaf	Dry	*Campylophyllum hispidulum*	55.6	34.3	8.83	0.025
Broadleaf	Dry	*Chiloscyphus pallescens*	22.2	9.9	5.66	0.066
Broadleaf	Dry	*Lophocolea heterophylla*	33.3	11.3	6.36	0.015
Broadleaf	Dry	*Plagiomnium drummondii*	42.9	26.1	9.50	0.057
Broadleaf	Dry	*Scapania glaucocephala*	27.5	13.2	6.44	0.057
Mixed	Wet	*Aulacomnium palustre*	45.0	27.8	10.09	0.064
Mixed	Wet	*Hypnum pratense*	23.5	11.9	6.18	0.095
Mixed	Wet	*Thuidium recognitum*	23.5	11.9	6.10	0.082
Mixed	Moist	*Hylocomium splendens*	53.1	40.8	6.81	0.055
Mixed	Moist	*Pleurozium schreberi*	65.2	41.4	9.11	0.018
Mixed	Dry	*Brachythecium campestre*	52.6	22.2	8.65	0.005
Mixed	Dry	*Elodium blandowii*	20.0	8.8	4.76	0.064
Mixed	Dry	*Orthotrichum obtusifolium*	34.9	12.9	5.89	0.008
Mixed	Dry	*Orthotrichum speciosum*	21.5	12.9	5.99	0.080
Mixed	Dry	*Plagiomnium drummondii*	28.4	16.6	7.12	0.091
Mixed	Dry	*Pylaisia polyantha*	34.0	20.5	8.05	0.062
Mixed	Dry	*Scapania glaucocephala*	29.1	13.8	6.36	0.038
Conifer	Wet	*Brachythecium* cf. *salebrosum*	28.6	14.7	5.51	0.024
Conifer	Wet	*Thuidium recognitum*	23.8	12.6	5.16	0.050
Conifer	Moist	*Dicranum fragilifolium*	36.5	22.5	6.70	0.050
Conifer	Moist	*Ptilidium pulcherrimum*	56.2	42.6	8.47	0.066


*Observed and randomized indicator values (IV) are reported for IV *P* < 0.10.*

## Discussion

Our analyses, partially supporting our expectations, revealed some level of association between DTW and bryophyte cover, richness, diversity, and composition, which emphasized the importance of plot-level variation in site moisture as a driver of bryophyte assemblages (Mills and Macdonald, 2005; Caners et al., 2010, 2013). Also as expected, we found that these relationships differed among broadleaf-, mixed and conifer-dominated forests.

Consistent with earlier studies (Nijland et al., 2015), broadleaf forests occupied the drier sites along the DTW gradient as compared to conifer-dominated forests, which tended to occupy the wetter end of the gradient. Broadleaf forests had very low bryophyte cover. In contrast to our expectations, there was no relationship of DTW with cover in either the broadleaf-dominated or mixed forest types, and richness and diversity were lower toward the wetter end of the site moisture gradient. Thus, while the drier sites in these forest types supported more bryophyte species, the higher diversity values indicated a rather even assemblage composed of species tolerant of drier conditions. The higher species richness on these sites may be due to less competition from large forest floor feathermosses, which generally had low abundances in these forest types. Feathermosses are often abundant under humid and shaded conditions in conifer forests (Frego and Carleton, 1995; Caners et al., 2013). Although competition among bryophytes is poorly understood (Rydin, 2009), feathermosses can colonize patches of forest floor or dead wood in later stages of decay (Crites and Dale, 1998; Mills and Macdonald, 2005), potentially displacing some of the many species that can grow on these substrates. The lack of relationship between bryophyte cover and DTW observed for the broadleaf and mixed forests could be largely attributable to the lower overall cover, which can be explained by factors such as unfavorable microclimates (e.g., high light intensities, higher litter pH, warm, and dry soil characteristics of broadleaf forest), coupled with smothering or chemical inhibitory effects of high inputs of broadleaf foliar litter (Startsev et al., 2008; Márialigeti et al., 2009). A thick layer of deciduous leaf litter imposes a physical barrier to the growth and establishment of forest floor bryophytes. Moreover, the accumulation and eventual decomposition of deciduous leaf litter, which is high in base cations, may increase nutrient availability in ways (e.g., nutrient toxicity) that reduce moss photosynthesis (Bubier et al., 2007) and competitive ability against vascular plants.

Conifer-dominated forests occupied the wetter end of the moisture gradient and, as expected, bryophyte cover was higher on the wetter sites within this forest type. Conifer forests had overall higher cover of bryophytes and the strong positive relationships with site wetness emphasized the importance of moister microsites for bryophytes in this forest type (Mills and Macdonald, 2004; Caners et al., 2013). Bryophyte assemblages in this forest type were dominated by species such as *Hylocomium splendens*, *Ptilium crista-castrensis*, *Ptilidium pulcherrimum*, and *Pleurozium schreberi* that have an affinity for moister habitats. On the contrary, species richness and diversity (indicating the level of dominance or unevenness in species abundance) were both lower on wetter sites. This suggested that, even though abundance was higher on the moist sites this consisted of an uneven assemblage (in terms of relative abundances of species) of fewer dominant species, especially feathermosses. In contrast, like for the broadleaf- and mixed forests, the drier sites in conifer forest had high evenness in the bryophyte assemblages, made up of species tolerant of dry conditions. This might be related to microhabitat distribution among wet and dry sites; for the latter, species with stricter microhabitat preferences might be more likely to be present in wet than in dry sites conditions.

Depth-to-water also explained significant variation in bryophyte species composition in mixed forests. The observed variation in species composition, as well as the results of indicator species analysis, indicated that bryophyte species differ in their affinity for site moisture conditions. For instance, species such as *Brachythecium campestre* and *Plagiomnium drummondii* and a few others preferred drier site conditions, while feathermosses such as *H*. *splendens* and *P*. *schreberi* were associated with moister sites. Quite notably, wetland species, such as *Sphagnum warnstorfii*, *Calliergon cordifolium*, *Drepanocladus* spp., *Rhizomnium pseudopunctatum*, *Leptodictyum riparium*, among others mostly occurred in the wet and moist site category in individual forests (Appendix S3), which attested to the validity of the DTW to capture species association with site wetness. However, many of these species did not show up strongly as indicator species as they were either uncommon or had very low abundances in the plots sampled. Many forest bryophytes are closely associated with particular moisture conditions (Bates, 1998; Wiklund and Rydin, 2004; Dynesius and Zinko, 2006) or specific substrates (Kuglerova et al., 2016). Understanding fine-scale moisture patterns across landscapes can provide information about habitat quality and species adapted to these conditions. Although bryophyte species affiliation to specific substrates was not examined in this study, unexplained variation in the relationships of bryophyte response attributes to the DTW index could be due to variation in substrate availability, which is an important determinant of bryophyte assemblages (Mills and Macdonald, 2004). A suggestion for future studies would be to determine if site wetness affects abundance, richness, and composition on specific substrates.

Finally, relationships of bryophyte cover, richness, diversity, and composition to DTW differed in terms of the ‘best’ flow-initiation threshold. Although models based on the different flow-initiation thresholds were functionally equivalent, our analysis determined that the 4 ha threshold resulted in the best supported model (or an equivalently good model) for the relationship of bryophyte response attributes to DTW in our study area. Lower (e.g., 0.5 ha) flow-initiation thresholds represent a landscape in which many areas would experience occasional surface wetness. In contrast, higher (e.g., 16 ha) thresholds give a more conservative estimate which predicts the landscape to be generally drier; thus plots at the wet end of the DTW gradient based on this threshold are very likely to be wet most of the time. However, the 4 ha flow-initiation threshold is suggested to coincide with the minimum catchment area for image-digitized or field-mapped stream channels with permanent or intermittent flow (Murphy et al., 2009). Our results based on the 4 ha flow-initiation threshold thus suggest that seasonal or inter-annual variation in site moisture or occasional wetting up may be important in understanding patterns of bryophyte richness, diversity, and composition.

## Conclusion

Recent advances in availability of, and processing methods for, remotely sensed data have created a new capacity to characterize terrain conditions and features using novel tools such as wet areas mapping. The results of this study, obtained from empirical analyses of remotely sensed LiDAR data and field-based vegetation data, demonstrate the prospects and utility of the topographic DTW index to help predict fine-scale patterns of bryophyte abundance, diversity, and composition in forested landscapes. Thus, rapid estimation of site moisture based on readily available remotely sensed LiDAR data can be a useful tool for the conservation of bryophyte diversity and their ecosystem functions. Our results suggest that, while wetter areas in conifer forests supported higher bryophyte cover, drier areas in all three forest types could be important for conserving infrequent species, even if they are present in low abundance. The association between DTW and bryophyte assemblages may be of practical importance when management decisions, such as site selection and choosing between potential harvest units require biodiversity considerations.

## Author Contributions

SM conceived and designed the study. SB, RC, and SM validated the data. JO and BW validated the wet-areas mapping processing. SB and SM analyzed the data. SB, RC, JO, BW, and SM wrote the paper.

## Conflict of Interest Statement

The authors declare that the research was conducted in the absence of any commercial or financial relationships that could be construed as a potential conflict of interest.

## Footnotes

1 http://www.emendproject.org
