# Supplementary material for: Relating Bryophyte Assemblages to a Remotely Sensed Depth-to-Water Index in Boreal Forests
Source: Front Plant Sci. 2018 Jun 25;9:858. doi: 10.3389/fpls.2018.00858 (PMC6026670; doi:10.3389/fpls.2018.00858)
Supplement: Supplementary file 1 [file Data_Sheet_1.docx]

# Supporting Information

### Appendix S1. Overview map of the EMEND study site in northwestern Alberta, Canada, showing the location of plots sampled in broadleaf, mixed, and conifer forest cover-types. Depth-to-water from the Wet-Areas Mapping (based on the 4 ha flow-initiation threshold) is shown in the background with darker shades signifying moist sites.


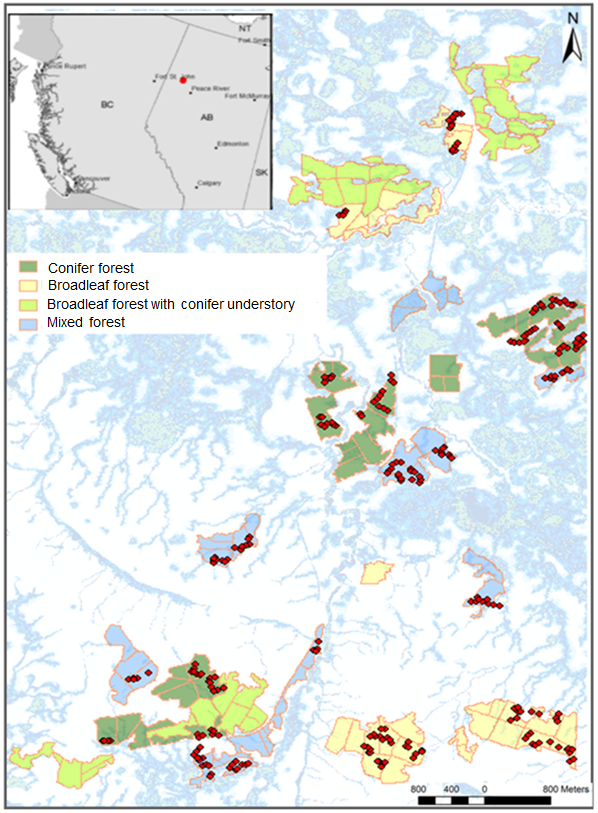


### Appendix S2. Species accumulation curves for the site wetness categories (wet [0–0.5 m], moist [0.6–2 m], and dry [> 2 m depth-to-water]) in broadleaf, mixed, and conifer forest cover-types. Note: species accumulation curve for dry site in conifer forest not shown because only one sample plot belonged in this category. The dark horizontal lines in the box and whisker plots represent the median, the ends of the box represent the lower and upper quartiles, the whiskers extend to the lowest and highest observations, and the separately plotted points are outliers.

### Appendix S3. List (in alphabetical order) and frequency (number of times a species occurred in plots associated with wet [0–0.5 m], moist [0.6–2 m], and dry [> 2 m depth-to-water] site conditions) of bryophyte species and their average abundance (% cover, in parentheses) in broadleaf-, mixed, and conifer-dominated forest cover-types. Species nomenclature for mosses follows the Flora of North America ([Flora of North America Editorial Committee](#_ENREF_21) 2007, [2014](#_ENREF_22)) and that for liverworts follows [Stotler and Crandall-Stotler (2017](#_ENREF_53)).

Form: M = moss, L = liverwort

| Bryophyte species | Code | Form | Broadleaf forest | | | Mixed forest | | | Conifer forest | | |
| --- | --- | --- | --- | --- | --- | --- | --- | --- | --- | --- | --- |
|  |  |  | wet  (*n* = 14) | moist  (*n* = 11) | dry  (*n* = 9) | wet  (*n* = 17) | moist  (*n* = 11) | dry  (*n* = 10) | wet  (*n* = 21) | moist  (*n* = 17) | dry  (*n* = 1) |
| *Amblystegium serpens* | Amb_ser | M | 12 (0.17) | 8 (0.07) | 8 (0.09) | 12 (0.24) | 4 (0.04) | 7 (0.07) | 4 (0.02) | 3 (0.02) | 1 (0.10) |
| *Aulacomnium palustre* | Aul_pal | M | 1 (0.01) |  | 1 (0.01) | 8 (1.08) | 2 (0.02) | 3 (0.03) | 6 (0.62) | 3 (0.04) | 1 (0.10) |
| *Blepharostoma trichophyllum* | Ble_tri | L | 2 (0.01) |  | 1 (0.01) | 1 (0.01) |  |  | 2 (0.01) | 1 (0.01) | 1 (0.10) |
| *Brachythecium acutum* | Bra_acu | M |  |  |  |  |  |  | 1 (0.14) |  |  |
| *Brachythecium* cf. *albicans* | Bra_alb | M |  |  |  |  |  |  | 1 (0.01) |  |  |
| *Brachythecium campestre* | Bra_cam | M | 7 (0.17) | 4 (0.11) | 5 (0.44) | 3 (0.07) | 2 (0.02) | 6 (0.63) | 5 (0.04) | 1 (0.01) |  |
| *Brachythecium* cf. *salebrosum* | Bra_sal | M | 5 (0.04) | 2 (0.02) | 1 (0.01) | 6 (0.40) | 3 (0.03) | 3 (0.07) | 6 (0.05) |  |  |
| *Brachytheciastrum velutinum* | Bra_vel | M |  | 1 (0.05) | 2 (0.02) |  | 2 (0.02) | 2 (0.02) | 1 (0.02) | 3 (0.02) |  |
| *Brachythecium* s.l. sp. | Bra_sp. | M | 7 (0.14) | 5 (0.05) | 6 (0.17) | 9 (0.38) | 5 (0.25) | 5 (0.05) | 5 (0.13) | 10 (0.08) | 1 (0.10) |
| *Bryum* s.l. sp. | Bry_sp. | M |  | 1 (0.01) |  | 2 (0.01) |  |  | 3 (0.01) |  |  |
| *Calliergon cordifolium* | Cal_cor | M |  |  |  | 2 (0.01) |  |  |  |  |  |
| *Campylophyllum hispidulum* | Cam_his | M | 7 (0.05) | 5 (0.08) | 8 (0.23) | 6 (0.21) | 3 (0.03) | 5 (0.05) | 2 (0.01) | 3 (0.02) | 1 (0.10) |
| *Ceratodon purpureus* | Cer_pur | M | 1 (0.01) | 2 (0.02) |  | 1 (0.01) | 1 (0.01) | 2 (0.02) | 2 (0.01) |  |  |
| *Chiloscyphus pallescens* | Chil_pal | L |  |  | 2 (0.02) |  | 1(0.01) | 1 (0.01) | 1 (0.01) |  |  |
| *Crossocalyx hellerianus* | Cro_hel | L |  |  |  |  | 2 (0.02) |  |  | 1 (0.01) | 1 (0.10) |
| *Dicranum acutifolium* | Dic_acu | M | 1 (0.01) |  |  |  |  |  | 3 (0.06) | 1 (0.01) |  |
| *Dicranum flagellare* | Dic_fla | M | 1 (0.01) |  | 1 (0.01) |  | 1 (0.01) |  |  | 1 (0.01) |  |
| *Dicranum fragilifolium* | Dic_fra | M |  |  |  | 3 (0.02) | 2 (0. 15) |  | 4 (0.02) | 7 (0. 15) | 1 (0.10) |
| *Dicranum fuscescens* | Dic_fus | M |  |  | 1 (0.01) | 1 (0.01) | 1 (0.01) | 1 (0.01) | 1 (0.01) | 2 (0.02) | 1 (0.10) |
| *Dicranum polysetum* | Dic_pol | M |  |  |  | 2 (0.04) | 2 (0.05) | 2 (0.02) | 1 (0.01) |  | 1 (0.10) |
| *Dicranum scoparium* | Dic_sco | M |  |  |  | 1 (0.01) | 1 (0.01) |  |  |  |  |
| *Dicranum undulatum* | Dic_und | M | 1 (0.01) |  |  |  |  | 1 (0.01) |  | 1 (0.01) |  |
| *Dicranum* sp. | Dic_sp. | M |  |  |  | 1 (0.01) | 1 (0.01) |  | 1 (0.02) | 1 (0.01) |  |
| *Drepanocladus aduncus* | Dre_adu | M |  | 2 (0.02) |  | 2 (0.01) |  |  | 2 (0.01) | 1 (0.01) |  |
| *Dreplanocladus polygamus* | Dre_pol | M | 1 (0.01) |  |  |  |  |  | 1 (0.01) |  |  |
| *Elodium blandowii* | Elo_bla | M |  |  |  |  |  | 2 (0.02) | 1 (0.02) |  |  |
| *Eurhynchiastrum pulchellum* | Eur_pul | M | 11 (0.19) | 5 (0.05) | 4 (0.24) | 11 (0.49) | 8 (0.11) | 8 (0.24) | 11 (0.11) | 9 (0.08) | 1 (0.50) |
| *Fuscocephaloziopsis lunulifolia* | Fus_lun | L |  |  |  |  | 1 (0.01) |  |  | 1 (0.01) |  |
| *Geocalyx graveolens* | Geo_gra | L |  |  |  |  |  |  |  | 2 (0.01) |  |
| *Haplocladium microphyllum* | Hap_mic | M |  | 1 (0.01) | 1 (0.01) | 1 (0.01) |  |  |  |  |  |
| *Herzogiella turfacea* | Her_tur | M |  |  |  | 2 (0.01) | 3 (0.03) | 1 (0.01) | 1 (0.01) | 2 (0.01) | 1 (0.10) |
| *Hygroamblystegium varium* var. *humile* | Hyg_var | M | 4 (0.03) | 1 (0.01) |  |  |  |  | 1 (0.01) |  |  |
| *Hylocomium splendens* | Hyl_spl | M | 6 (1.24) | 2 (0.19) | 2 (0.02) | 13 (6.14) | 10 (17.10) | 10(6.02) | 20 (20.96) | 17 (18.0) | 1 (0.10) |
| *Hypnum pratense* | Hyp_pra | M |  |  | 1(0.01) | 4 (0.07) |  |  | 3 (0.03) |  |  |
| *Isopterygiopsis pulchella* | Iso_pul | M |  |  |  | 2 (0.01) |  | 1 (0.01) |  |  | 1 (0.10) |
| *Lepidozia reptans* | Lep_rep | M |  |  |  |  |  |  | 1 (0.01) | 1 (0.01) |  |
| *Leptobryum pyriforme* | Lep_pyr | M | 1 (0.01) |  |  | 1 (0.01) |  |  | 2 (0.01) |  |  |
| *Leptodictyum riparium* | Lep_rip | M | 3 (0.02) | 2 (0.02) | 3 (0.03) | 3 (1.47) |  |  |  | 2 (0.01) |  |
| *Lophocolea bidentata* | Lop_bid | L |  |  |  |  | 1 (0.01) |  |  |  |  |
| *Lophocolea heterophylla* | Lop_het | L |  |  | 3 (0.03) | 3 (0.04) | 1 (0.01) | 2 (0.02) | 1 (0.01) |  | 1 (0.10) |
| *Lophocolea minor* | Lop_min | L | 1 (0.01) |  |  | 3 (0.02) | 1 (0.01) |  | 3 (0.01) | 1 (0.01) |  |
| *Lophocolea* sp. | Lop_sp. | L |  | 1 (0.01) |  |  |  | 1 (0.01) |  |  |  |
| *Lophozia ascendens* | Lop_asc | L |  |  |  |  | 1 (0.01) | 1 (0.01) | 1 (0.01) |  | 1 (0.10) |
| *Lophozia* s.l. *ventricosa* | Lop_ven | L |  |  |  |  |  |  |  |  | 1 (3.0) |
| *Lophozia* sp. | Lop_sp. | L |  |  |  |  |  |  |  | 1 (0.01) | 1 (0.01) |
| *Lophoziopsis excisa* | Lop_exc | L |  |  |  |  |  |  |  | 1 (0.01) |  |
| *Lophoziopsis longidens* | Lop_lon | L |  |  |  |  | 1 (0.01) |  |  |  |  |
| *Mnium spinolosum* | Mni_spi | M | 1 (0.01) |  |  | 2 (0.04) | 3 (0.03) | 4 (0.08) | 3 (0.01) | 5 (0.03) |  |
| *Mnium marginatum* | Mni_mar | M | 1 (0.01) |  |  | 1 (0.01) |  |  |  |  |  |
| *Oncophorus wahlenbergii* | Onc_wah | M | 4 (0.03) | 2 (0.02) | 2 (0.02) | 6 (0.04) | 3 (0.03) | 4 (0.04) | 5 (0.07) | 2 (0.01) | 1 (0.10) |
| *Orthotrichum obtusifolium* | Ort_obt | M | 3 (0.02) | 3 (0.06) | 4 (0.04) | 1 (0.01) |  | 4 (0.04) |  | 1 (0.01) |  |
| *Orthotrichum speciosum* | Ort_spe | M | 2 (0.01) | 3(0.03) | 2 (0.02) | 2 (0.01) |  | 3 (0.03) |  |  |  |
| *Plagiomnium cuspidatum* | Pla_cus | M | 5 (0.09) | 5 (0.08) | 6 (0.17) | 1 (0.01) | 2 (0.28) | 6 (0.20) | 2 (0.01) |  | 1 (0.10) |
| *Plagiomnium drummondii* | Pla_dru | M | 5 (0.04) | 1 (0.01) | 5 (0.16) |  | 2 (0.02) | 3 (0.32) | 2 (0.02) | 1 (0.01) | 1 (0.10) |
| *Plagiomnium ellipticum* | Pla_ell | M | 4 (0.23) | 3 (0.03) | 1 (0.01) | 7 (0.12) | 1 (0.05) | 1 (0.01) | 7 (0.30) | 6 (0.15) |  |
| *Plagiomnium medium* | Pla_med | M | 2 (0.01) | 2 (0.02) | 2 (0.02) | 2 (0.01) | 3 (0.03) | 2 (0.02) | 4 (0.06) | 1 (0.01) | 1 (0.10) |
| *Plagiomnium* sp. | Pla_sp. | M | 9 (0.19) | 5 (0.29) | 4 (0.37) | 10 (1.71) | 2 (0.73) | 2 (0.02) | 9 (0.52) | 3 (0.04) |  |
| *Plagiothecium laetum* | Pla_lae | M |  |  |  |  |  |  |  | 1 (0.01) |  |
| *Plagiothecium denticulatum* | Pla_den | M |  | 1 (0.01) |  | 4 (0.08) |  |  |  |  |  |
| *Plagiothecium* cf. *latebricola* | Pla_lat | M |  |  |  | 1 (0.06) |  |  |  |  |  |
| *Plagiochila porelloides* | Pla_por | L | 1 (0.01) |  |  | 2 (0.01) |  |  | 2 (0.01) |  |  |
| *Pleurozium schreberi* | Ple_sch | M | 4 (0.24) | 1 (0.01) | 2 (0.02) | 10 (0.85) | 10 (6.75) | 10(1.81) | 19(4.04) | 16 (3.84) | 1 (1.50) |
| *Pohlia nutans* | Poh_nut | M |  | 2 (0.05) | 1 (0.01) | 5 (0.03) | 4 (0.04) | 2 (0.02) | 3 (0.01) | 4 (0.02) | 1 (0.10) |
| *Pohlia wahlenbergii* | Poh_wah | M | 1 (0.01) |  |  |  |  |  |  |  |  |
| *Polytrichum commune* | Pol_com | M |  |  |  |  |  | 1 (0.01) |  | 1 (0.01) |  |
| *Polytrichum juniperinum* | Pol_jun | M |  |  |  |  |  |  | 1 (0.01) |  |  |
| *Ptilidium pulcherrimum* | Pti_pul | L | 4(0.03) | 1 (0.01) | 1 (0.01) | 5(0.05) | 6 (0.26) | 4 (0.04) | 8 (0.06) | 11 (0.38) | 1 (0.10) |
| *Ptilium crista-castrensis* | Pti_cri | M | 3 (0.02) | 1 (0.01) | 2 (0.02) | 9 (0.36) | 9 (3.39) | 7 (4.86) | 19(5.11) | 16 (5.88) | 1 (1.0) |
| *Ptychostomum creberrimum* | Pty_cre | M | 1 (0.01) | 2 (0.02) |  |  |  | 1 (0.01) | 2(0.03) |  |  |
| *Ptychostomum pseudotriquetrum* | Pty_pse | M | 1 (0.01) |  |  | 1 (0.01) |  |  |  |  |  |
| *Pylaisia polyantha* | Pyl_pol | M | 9 (0.29) | 7 (0.45) | 6 (0.21) | 4 (0.08) |  | 6 (0.10) | 3 (0.06) | 4 (0.10) | 1 (0.10) |
| *Rhizomnium pseudopunctatum* | Rhi_pse | M | 1 (0.04) |  |  | 1 (0.24) |  |  | 2 (0.20) |  |  |
| *Rhizomnium* sp. | Rhi_sp. | M |  |  |  | 1 (0.01) |  |  |  |  |  |
| *Riccardia latifrons* | Ric_lat | L |  |  |  |  |  |  | 1(0.01) |  |  |
| *Riccardia* sp. | Ric_sp. | L |  |  |  | 1 (0.01) |  |  |  | 1 (0.01) | 1 (0.10) |
| *Sanionia uncinata* | San_unc | M | 7 (0.05) | 4 (0.04) | 5 (0.16) | 12 (0.12) | 5 (0.08) | 7 (0.15) | 7 (0.03) | 4 (0.02) | 1 (0.10) |
| *Scapania glaucocephala* | Sca_gla | L | 1 (0.01) |  | 3 (0.03) | 1 (0.01) | 1 (0.01) | 4 (0.04) | 1 (0.01) | 1 (0.01) |  |
| *Sciuro-hypnum curtum* | Sci_cur | M | 3 (0.05) | 2 (0.05) | 2 (0.12) | 9 (0.49) | 3 (0.11) | 6 (0.45) | 10(0.15) | 7 (0.06) |  |
| *Sciuro-hypnum reflexum* | Sci_ref | M |  |  |  | 1 (0.01) |  |  |  |  | 1 |
| *Sphagnum capillifolium* | Sph_cap | M |  |  |  | 1 (0.18) |  |  |  |  |  |
| *Sphagnum warnstorfii* | Sph_war | M |  |  |  | 2 (0.21) |  |  | 2 (0.03) |  |  |
| *Syzygiella autumnalis* | Syz_aut | L | 1 (0.01) |  | 2 (0.02) | 2 (0.01) | 2 (0.02) | 3 (0.03) | 2 (0.01) | 6 (0.04) | 1 (0.10) |
| *Tetraphis pellucida* | Tet_pel | M |  |  |  |  |  |  |  | 1 (0.01) |  |
| *Tetraplodon angustatus* | Tet_ang | M |  |  |  |  |  | 1 (0.01) |  |  |  |
| *Thuidium recognitum* | Thu_rec | M | 1 (0.01) |  | 2 (0.02) | 4 (0.07) |  |  | 5 (0.06) |  | 1 (0.10) |
| *Tomentypnum nitens* | Tom_nit | M |  |  |  | 4 (0.12) |  | 1 (0.01) | 2 (0.03) |  |  |

### Appendix S4. Comparison of AICcs and ∆AICc of linear mixed effect models for the relationship between depth-to-water (DTW) and bryophyte cover, richness, and diversity at different flow-initiation thresholds. For the most supported model (lowest AICc) for each response variable, the AICc is highlighted in bold. Shaded font values correspond to the flow-initiation thresholds for which ∆AICc < 2 across all three response variables (bryophyte cover, richness, and diversity); based on this we chose an initiation threshold of 4 ha for all models.

| Threshold | AICc | | | ∆AICc | | |
| --- | --- | --- | --- | --- | --- | --- |
|  | Cover | Richness | Diversity | Cover | Richness | Diversity |
| **A.** Broadleaf forest | | | | | | |
| 0.5 ha | **94.62** | 263.17 | 181.81 | 0 | 12.32 | 2.02 |
| 1 ha | 96.12 | 254.88 | 181.05 | 1.49 | 4.02 | 1.26 |
| 2 ha | 96.34 | 253.65 | 181.23 | 1.71 | 2.80 | 1.44 |
| 4 ha | 96.57 | **250.86** | **178.80** | 1.95 | 0 | 0 |
| 8 ha | 96.77 | 252.5 | 182.07 | 2.14 | 1.65 | 2.28 |
| 12 ha | 95.71 | 263.75 | 186.65 | 1.08 | 12.89 | 6.85 |
| 16 ha | 96.74 | 263.32 | 186.5 | 2.12 | 12.47 | 6.71 |
| **B.** Mixed forest | | | | | | |
| 0.5 ha | **151.67** | 303.6 | 80.23 | 0 | 7.21 | 2.02 |
| 1 ha | 152.46 | 305.76 | 79.66 | 0.79 | 9.37 | 1.45 |
| 2 ha | 152.94 | 301.65 | **78.21** | 1.27 | 5.27 | 0 |
| 4 ha | 153.33 | 296.74 | 78.32 | 1.66 | 0.34 | 0.11 |
| 8 ha | 153.36 | **296.39** | 78.49 | 1.69 | 0 | 0.28 |
| 12 ha | 153.32 | 296.68 | 78.76 | 1.64 | 0.28 | 0.56 |
| 16 ha | 153.29 | 296.49 | 79.01 | 1.61 | 0.10 | 0.80 |
| **C.** Conifer forest | | | | | | |
| 0.5 ha | 170.42 | 268.03 | 50.79 | 0.92 | 1.93 | 7.12 |
| 1 ha | 170.93 | 269.08 | 43.81 | 1.43 | 2.98 | 0.14 |
| 2 ha | 170.99 | 268.25 | **43.67** | 1.49 | 2.15 | 0 |
| 4 ha | 170.71 | **266.1** | 44.97 | 1.21 | 0 | 1.3 |
| 8 ha | 171.97 | 273.78 | 51.66 | 2.46 | 7.67 | 7.99 |
| 12 ha | 170.35 | 275.15 | 50.57 | 0.85 | 9.04 | 6.9 |
| 16 ha | **169.5** | 269.86 | 51.94 | 0 | 3.76 | 8.27 |

### **Appendix S5**.Correlation (product-moment correlation coefficients) between the different flow-initiation thresholds used for the calculation of depth-to-water in broadleaf, mixed, and conifer forest-cover types. Significant correlation coefficients (α = 0.05) are denoted by asterisks (*).

| Forest type | Flow-initiation threshold | | | | | |
| --- | --- | --- | --- | --- | --- | --- |
|  | 0.5 ha | 1 ha | 2 ha | 4 ha | 8 ha | 12 ha |
| **A.** Broadleaf forest | | | | | | |
| 0.5 ha |  |  |  |  |  |  |
| 1 ha | 0.75* |  |  |  |  |  |
| 2 ha | 0.70* | 0.99* |  |  |  |  |
| 4 ha | 0.63* | 0.94* | 0.97* |  |  |  |
| 8 ha | 0.36* | 0.75* | 0.77* | 0.81* |  |  |
| 12 ha | 0.27 | 0.42* | 0.42* | 0.44* | 0.52* |  |
| 16 ha | 0.19 | 0.34* | 0.35* | 0.38* | 0.50* | 0.93* |
|  |  |  |  |  |  |  |
| **B.** Mixed forest | | | | | | |
| 0.5 ha |  |  |  |  |  |  |
| 1 ha | 0.92* |  |  |  |  |  |
| 2 ha | 0.70* | 0.82* |  |  |  |  |
| 4 ha | 0.43* | 0.52* | 0.88* |  |  |  |
| 8 ha | 0.41* | 0.52* | 0.87* | 0.99* |  |  |
| 12 ha | 0.39* | 0.51* | 0.87* | 0.99* | 0.99* |  |
| 16 ha | 0.37* | 0.50* | 0.85* | 0.97* | 0.99* | 0.99* |
|  |  |  |  |  |  |  |
| **C.** Conifer forest | | | | | | |
| 0.5 ha |  |  |  |  |  |  |
| 1 ha | 0.59* |  |  |  |  |  |
| 2 ha | 0.56* | 0.99* |  |  |  |  |
| 4 ha | 0.54* | 0.97* | 0.97* |  |  |  |
| 8 ha | 0.01 | 0.07 | 0.08 | 0.14 |  |  |
| 12 ha | 0.001 | 0.06 | 0.06 | 0.13 | 0.98* |  |
| 16 ha | -0.09 | -0.02 | -0.02 | 0.04 | 0.94* | 0.96* |
